# Reply to Bareille et al. Are Viscoelastometric Assays of Old Generation Ready for Disposal? Comment on “Volod et al. Viscoelastic Hemostatic Assays: A Primer on Legacy and New Generation Devices. *J. Clin. Med.* 2022, *11*, 860”

**DOI:** 10.3390/jcm12020478

**Published:** 2023-01-06

**Authors:** Oksana Volod, Connor M. Bunch, Joseph Miller, Ernest E. Moore, Hunter B. Moore, Hau C. Kwaan, Shivani S. Patel, Grant Wiarda, Michael Aboukhaled, Scott G. Thomas, Daniel Fulkerson, Lee Erdman, Anna Tincher, Mark M. Walsh

**Affiliations:** 1 Department of Pathology and Laboratory Medicine, Cedars-Sinai Medical Center, Los Angeles, CA 90048, USA; 2 Department of Emergency Medicine, Henry Ford Hospital, Detroit, MI 48402, USA; 3 Department of Internal Medicine, Saint Joseph Regional Medical Center, Mishawaka, IN 46545, USA; 4 Department of Surgery, Ernest E. Moore Shock Trauma Center at Denver Health, University of Colorado Health Sciences Center, Denver, CO 80204, USA; 5 Division of Hematology and Oncology, Department of Medicine, Northwestern University Feinberg School of Medicine, Chicago, IL 60611, USA; 6 Department of Trauma Surgery, Memorial Leighton Trauma Center, Beacon Health System, South Bend, IN 46601, USA

We are pleased to see that Bareille et al. have written a Commentary: “Are viscoelastometric assays of old generation ready for disposal?” [1] in response to our paper published in the Special Edition of the Journal of Clinical Medicine by Volod et al. [2].

The authors have expressed concern regarding our perceived excessive enthusiasm for the utilization of VHAs to define hemostatic derangement in many clinical settings and for what they call the “so called” COVID Associated Coagulopathy (CAC). We feel that it is necessary to provide a rebuttal to their commentary.

This rebuttal focuses on two points.

The first concerns Bareille et al.’s disagreement with our emphasis on the term COVID Associated Coagulopathy (CAC).

The second point addresses Bareille et al.’s objection that we have failed to point out that these legacy and post-legacy VHA devices are not “interchangeable”.

Regarding the first point, the authors referred to our review of the use of VHAs in COVID-19 and our use of the term “coagulopathy” to define the universally observed proclivity of these patients to not only present with clotting anomalies but also to simultaneously hemorrhage. They object to our use of the term “coagulopathy” and they suggest replacing it with their term of “disordered hemostasis”. They further note that this “disordered hemostasis” can often cause patients to “exhibit both thrombotic and hemorrhagic complications”. They express concern that we did not address this duality. In fact, we mentioned this phenomenon that uniquely defines COVID-19 patients. In our manuscript on page 13, we specifically state that these patients can “clot and bleed at the same time” thus agreeing with Bareille et al.’s characterization of the singular nature of the “disordered hemostasis” in COVID-19 patients. During the peak two years of the COVID-19 pandemic, our group and others relied heavily on adjunctive VHAs to guide the anticoagulation of patients who had a COVID-associated coagulopathy or “disordered homeostasis”, as Barielle et al. would prefer to call this unique hemostatic derangement. However, the term CAC has been used by many hematologic societies to describe the unique characteristic of these patients to “clot and bleed at the same time” [3,4,5,6,7,8,9].

Whether the term “CAC” or “disordered hemostasis” is used to define this unique characteristic of COVID-19 patients to “clot and bleed at the same time”, as we have written in our paper, matters little as we and many others have used VHAs to thread the needle and avoid the complications of under- and over-coagulation at either end of the hemostatic spectrum of the “so called” CAC or “disordered hemostasis” as they describe it [10]. Given the demonstrated benefit of VHAs to guide heparin anticoagulation, the FDA approved VHAs specifically for the purpose to assist clinicians in this difficult task of anticoagulating patients with CAC [11]. Clearly, CAC is a singular and authentic entity where patients with fibrinolysis shutdown clot and bleed at the same time [12,13].

Finally, a recent review of the literature using the MeSH term COVID associated coagulopathy reveals that this term was a standard descriptor for the singular observation of these patients to develop early clots that required delicate and closely monitored anti-coagulation [8,14,15,16,17,18,19,20,21,22,23,24,25,26,27,28,29,30,31,32,33,34,35,36,37].

Our second point concerns Bareille et al.’s statement that we have not emphasized enough the lack of interchangeability of the legacy and post-legacy devices. We state the following regarding the interchangeability of the TEG 5000 and the TEG6s on page 4, Table 1: “For the TEG^®^ 6s and the TEG^®^ 5000, the manufacturer (Haemonetics, Braintree, Massachusetts) states that any difference in the values is a consequence of the Clinical and Laboratory Standards Institute (CLSI) methodology used for the TEG^®^ 6s values. Additionally, the manufacturer’s ranges are not globally prescriptive, as every laboratory must establish their own reference intervals. The values for clinically significant fibrinolysis vary greatly in the literature”.

They point out that the older as well as the post-legacy new generation VHAs are not interchangeable. We agree. We clearly state on page 5 the following in our paper regarding comparison of these two devices: “...the thresholds for intervention are not uniform and depend on local standards that determine the triggers for intervention. VHA guidance of BCTs, and pro-hemostatic agents with simplified algorithms, facilitate goal-directed BCT and HAT for bleeding and anticoagulated patients”. We specifically address the idea of interchangeability with the following on page 6: “although the TEG^®^ 6s and the TEG^®^ 5000 use slightly different mechanisms to assess similar coagulation components, there is a small difference in absolute values of respective parameters of the two devices. Therefore, the absolute values are not interchangeable”.

The authors are to be commended for their extensive review of the literature of the latest post-legacy generation devices and for their very complete table, which bolsters their argument as well as ours for the lack of interchangeability of these new devices. This table will be a useful instrument for those who wish to compare the efficacy of these devices much as our Table 3 on page 7, which compared the same devices to the cup and pin methods, will be. However, it remains the case that our Table 3 and the authors’ table comparing the devices again manifest what has been a central tenet in the literature since the inception of the VH: that these VHA devices, whether legacy or post-legacy, cannot be used in a comparative setting and that each institution must establish its own standardization. In our legend for Table 3 in our manuscript, where we broadly describe the various types of legacy and post-legacy VHAs in our “primer” on VHAs, we refer to the literature that clearly states that the parameters for these many devices are not interchangeable.

On a technical point, regarding the comparison of legacy and post-legacy devices, the authors assert that we do not identify Sonoclot as a legacy system. However, we state on page 5: “The TEG^®^ 5000 and the ROTEM^®^ delta systems are considered cup and pin “legacy” systems. Another legacy linear motion system is called Sonoclot^®^; however, it is not a rotation based system”.

In summary, we are very grateful for the commentary that Bareille et al. have given on our manuscript and we point out that for the special edition of the JCM devoted to VHAs our “primer” paper by Volod et al. was of central importance because of its purpose of providing an introduction for experts and nonexperts to viscoelastic testing in the clinical and research settings. We believe that Bareille et al.’s characterization of our enthusiasm as excessive contradicts the extraordinary experience and growth of these devices not just during the pandemic but also recently in the care of severe trauma patients and in patients with post-partum hemorrhage, (PPH), extracorporeal mechanical oxygenation (ECMO) as well as in surgical and medical critical care medicine [38,39,40,41,42,43,44,45,46].

The authors have also invoked the objection that before the widespread use of these VHA devices is undertaken we need larger RCTs to confirm their worth. We would like to point out that the activated patrial thromboplastin time (aPTT) test was developed around the time of TEG. If one peruses how aPTT and heparin therapeutic ranges were established, they would be surprised by the lack of large RCTs that established the norms for guiding heparin anticoagulation. Nevertheless, standard algorithms have been universally adopted by hematologists and other specialties.

In fact, the “1.5 to 2.5 times control” UFH therapeutic range was established is instructive regarding the history of RCTs in offering guidance for heparin anticoagulation. In 1972, a lower risk of recurrent thromboembolism occurrence among patients anticoagulated with heparin within an aPTT of 1.5 to 2.5 times the mean of the laboratory reference range was reported in the original retrospective analysis by Basu et al. [47]. An experimental rabbit model of thrombus extension supported the 1.5 to 2.5 therapeutic range in a subsequent study using results with the same aPTT reagents [48]. However, randomized controlled trials have not confirmed the clinical relevance of this therapeutic range. However, using a protamine–heparin titration assay and 0.35 to 0.70 IU/mL with a factor Xa assay, an aPTT of 1.5 to 2.5 times control was found to be equivalent to a heparin level of 0.2 to 0.4 IU/mL [49]. Levine et al. formed the basis for the recommendation of a 0.3 to 0.7 IU/mL therapeutic range for UFH using an anti-Xa assay by demonstrating this relationship in a randomized controlled trial study [45,49,50].

The more recent literature confirms the lack of large RCTs supporting specific algorithms regarding the aPPT or anti-Xa levels to guide heparin therapy. An authoritative review concerning guidance for the practical management of the heparin anticoagulants in the treatment of venous thromboembolism in 2016 stated that: “Despite the standard practice to monitor heparin through coagulation laboratory testing, the body of evidence supporting monitoring is surprisingly weak”. As a result, the guidelines and algorithms are still in a state of evolution [51].

Bareille et al. also harken to the use of “rapid coagulation tests” as a substitute for the VHAs without noting that the very citations they use for these rapid tests (which claim the capability of obtaining actionable results in 20 min) have not been successfully reproduced despite the antiquated references by the authors dating from 2010 and 2013 [52,53].

Precision-based medicine (PBM) adopts the concept of describing a hemostatic phenotype defined by VHA and common coagulation tests (CCTs) to guide anticoagulation and blood component therapy (BCT) and hemostatic adjunctive therapy (HAT) in many clinical situations. The “one size fits all” methodology of awaiting large RCTs to confirm the validity of VHAs would have deprived the medical community of the rapid advances that have occurred during the pandemic and that led to the early approval of VHAs by the FDA for use in guiding the anticoagulation of COVID-19 patients [11,38,54,55,56].

We agree with the authors that large RCTs can be complementary in affirming the clinical utility of VHAs (provided appropriate data quality control). During the pandemic, clinicians who relied on an evidence-based medicine approach were forced to practice with evolving data concerning the escalation of anticoagulation in the COVID-19 patients who clearly presented with a very high rate of thrombosis [57]. Therefore, during this early period of the pandemic without the benefit of RCTs many institutions and hematologic societies provided diverse and evolving guidelines regarding the escalation of prophylactic anticoagulation to intermediate or therapeutic doses of heparin for patients with COVID-19 pneumonitis without macrovascular thrombosis, often relying on VHAs to assist in guiding the anticoagulation of these patients who, as we stated, would “clot and bleed at the same time” [58,59,60,61,62,63,64,65,66,67,68,69,70,71,72,73,74].

However, as the COVID-19 pandemic has illustrated, the reliance on hypotheses generated by smaller studies during times of epidemiologic uncertainty has provided us with a unique opportunity to advance the cause of precision-based guidance for the treatment of patients with not just COVID-19-related coagulopathies but of all severe coagulopathies related to liver transplantation, cardiac surgery, trauma resuscitation, ECMO, PPH and other areas of medical and surgical coagulopathy where the assessment of a patient’s hemostatic phenotype requires not just CCTs but also VHA analysis [44].
